# ^68^Ga-labeled amphiphilic polymer nanoparticles for PET imaging of sentinel lymph node metastasis

**DOI:** 10.1093/rb/rbad029

**Published:** 2023-03-27

**Authors:** Qin Chen, Xiaomin Fu, Huawei Cai, Shengxiang Fu, Zhongyuan Cai, Mufeng Li, Xiaoai Wu, Rong Tian, Hua Ai

**Affiliations:** National Engineering Research Center for Biomaterials, Sichuan University, Chengdu 610064, China; National Engineering Research Center for Biomaterials, Sichuan University, Chengdu 610064, China; Department of Nuclear Medicine, West China Hospital, Sichuan University, Chengdu 610041, China; National Engineering Research Center for Biomaterials, Sichuan University, Chengdu 610064, China; National Engineering Research Center for Biomaterials, Sichuan University, Chengdu 610064, China; Department of Nuclear Medicine, West China Hospital, Sichuan University, Chengdu 610041, China; Department of Nuclear Medicine, West China Hospital, Sichuan University, Chengdu 610041, China; Department of Nuclear Medicine, West China Hospital, Sichuan University, Chengdu 610041, China; National Engineering Research Center for Biomaterials, Sichuan University, Chengdu 610064, China; Department of Radiology, West China Hospital, Sichuan University, Chengdu 610041, China

**Keywords:** amphiphilic alternating copolymers, rigidity, PET, lymph node metastasis, labeling efficiency

## Abstract

Precise diagnosis of lymph node metastasis is important for therapeutic regimen planning, prognosis analysis and probably better outcomes for cancer patients. In this work, ^68^Ga-labeled amphiphilic alternating copolymers nanoparticles with different rigid ligands were synthesized as positron emission tomography (PET) probes for lymph node metastasis imaging. The labeling efficiency and stability of nanoparticles was improved with increased rigidity of coordination unit. PU(^68^Ga-L-MDI-PEG) nanoparticles (PU(^68^Ga-L-MDI-PEG) NPs) with the strongest rigidity of coordination unit exhibited the lowest critical micelle concentration, the best ^68^Ga labeling efficiency and stability. During *in vivo* lymph node metastasis imaging, PU(^68^Ga-L-MDI-PEG) NPs led to different accumulations in normal lymph nodes (N-LN) and tumor metastasized sentinel lymph nodes (T-SLN), which resulted in different PET signal presentation, making it feasible to differentiate N-LN from T-SLN. In comparison, small molecule probe ^68^GaL had poor lymph node accumulation, not only making it difficult to find lymph nodes on PET/computed tomography scan, but also tough to distinguish N-LN from metastatic ones. Overall, this work provides a reference for design of ^68^Ga labeled polymeric nanoparticles with high chelation efficiency and stability, as sensitive PET probes for lymph node imaging.

## Graphical Abstract

**Figure:**
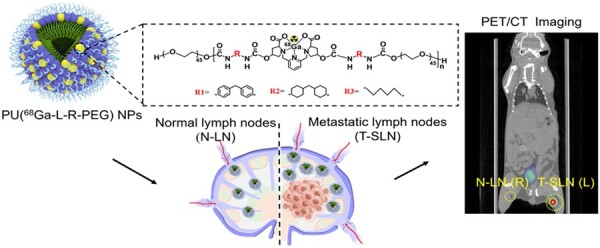

## Introduction

Tumor metastasis is one of the main causes of tumor-related death [1, 2]. Sentinel lymph node (SLN) is the first lymph node that tumor drainage reaches [3–5]. If SLN is diagnosed as positive, it often indicates a high incidence of distant metastasis, poor prognosis and high recurrence rate [6, 7]. Therefore, the diagnosis of SLN metastasis is of great clinical significance for tumor staging and treatment decisions. Many medical imaging techniques have been used to evaluate lymph node status clinically, such as computed tomography (CT), positron emission tomography (PET), fluorescence and magnetic resonance imaging [8–11]. Among them, PET imaging with high sensitivity and excellent depth penetration has unique advantages for lymph node imaging. ^18^F-fluorodeoxyglucose (^18^F-FDG), a glucose analog, is the most widely used PET tracer in clinical lymph node imaging [12, 13]. The glucose metabolism of the invaded lymph nodes always increases, leading to increased uptake of ^18^F-FDG and enhanced radioactive signals. However, small molecule ^18^F-FDG has some disadvantages, such as fast clearance kinetics, short imaging time window and so on [14, 15].

In order to ameliorate these problems, researchers have developed nanosized contrast agent for lymph node imaging with better imaging time window and signal enhancement. According to literature reports, the size, zeta-potential and surface modification of nano-contrast agent play an important role in lymphatic transmission and lymph node retention. The ideal lymph node contrast agent is a negative charged nano-contrast agent with the size of 10–100 nm [16, 17]. However, there are few studies on the application of PET nano-contrast agent in lymph node imaging. Therefore, it is of great significance to develop a PET probe and explore its application in lymph node metastasis imaging [18].

**Scheme 1.**
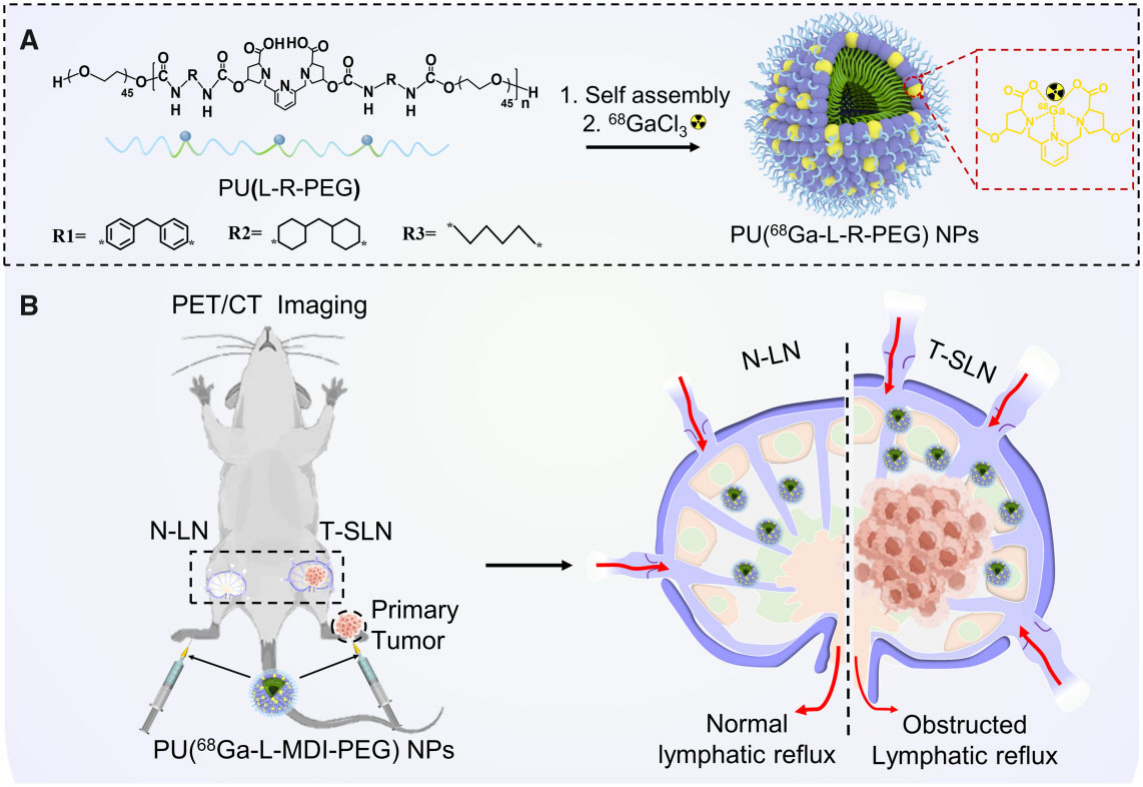
Schematic diagram of ^68^Ga-labeled amphiphilic polymer nanoparticles and PET imaging lymph node metastasis. (**A**) Synthesis of amphiphilic alternating copolymer nanoparticles (PU(^68^Ga-L-R-PEG) NPs, R = R1, R2 and R3). (**B**) PU(^68^Ga-L-MDI-PEG) NPs were used for PET/CT imaging of mice with lymph node metastases. N-LN, normal lymph nodes; T-SLN, metastatic lymph nodes.

Radioactive metals usually chelate with small molecular ligands (such as DOTA) to form stable complexes as PET probes. However, some ligands with low chelation stability may cause inaccurate imaging and a possible risk of toxicity due to the dechelation of radioactive metal ions. According to previous reports, the rigidity of coordination unit has an important impact on coordination stability [19–22]. Therefore, three prepolymers with different rigidity of coordination units were prepared by prepolymerization of three isocyanates and small molecules with the structure of an aza-semisolid ether. We speculate that with the increase of rigidity of coordination unit, the rotation of ligand molecules is limited, which may improve their chelating stability. Then, different rigid prepolymers were polymerized with polyethylene glycol to obtain amphiphilic alternating copolymers and self-assembled into nanoparticles (NPs) [23]. Finally, the PET imaging probe was obtained by labeling nanoparticles with ^68^Ga and used to explore the potential of lymph node metastasis by PET imaging (Scheme 1).

## Experimental

### Materials

L-hydroxyproline (99%), L-proline (99%), thionyl chloride (99.5%), 2,6-pyridine dichloride (98%), citric acid, sodium acetate and sodium hydroxide (97%) were purchased from Aladdin (Shanghai, China), 4-4′-dicyclohexylmethane diisocyanate (HMDI; 90.0%), hexamethylene diisocyanate (HDI; 99%), 4,4′-diphenylmethane diisocyanate (MDI; 98%), butyltin dilaurate (95%) and mPEG_2k_ were bought from TCI (Shanghai) Chemical Industry Development Corporation. Column chromatography field silica gel was purchased from Qingdao Ocean Chemical Co., Ltd, 200–300 mesh; All reagents used in this work were analytical or higher grade.

### Synthesis and characterization of small molecule complexes (L)

Small molecule complexes were synthesized according to previous laboratory work [24].

### Synthesis of amphiphilic alternating copolymers PU(L-R-PEG)

#### Synthesis of PU(L-MDI-PEG) precursor

Compounds 1 (1 mmol) and MDI (0.5 mmol) were dissolved in anhydrous *N*, *N*-dimethylformamide (DMF; 1.5 ml), respectively, and a prepolymer was formed at 25°C for 3 h. mPEG_2k_ (0.5 mmol) dissolved in 1.5 ml of anhydrous DMF was then added and reacted for 38 h. Then, mPEG_2k_ (0.11 mmol) was added to the above solution for 10 h to end-capping reaction. Finally, the mixture was precipitated with ether three times and dialyzed with deionized water for three days. The precursor of PU(L-MDI-PEG) was obtained by freeze-drying. Yield: 1.3 g (81%).

#### Synthesis of PU(L-MDI-PEG)

The PU(L-MDI-PEG) precursor (1.3 g) was dissolved in a mixed solution of 7 ml methanol and tetrahydrofuran (THF; volume ratio 1:6), and 3 ml NaOH (0.1 M) aqueous solution was added stirring at 25°C for 24 h, and then dialyzed with distilled water for 3 days. Finally, the product was acquired by lyophilization.

The other two amphiphilic alternating copolymers PU(L-HMDI-PEG) and PU(L-HDI-PEG) were obtained under the same reaction condition as PU(L-MDI-PEG), and except that isocyanates (MDI) was replaced by two different rigid isocyanates (HMDI and HDI).

#### Synthesis of amphiphilic alternating copolymer nanoparticles (PU(L-R-PEG) NPs)

PU(L-R-PEG) (0.1 g) dissolved in 0.5 ml dimethyl sulfoxide (DMSO) was added drop by drop to 20 ml deionized water to obtain PU(L-R-PEG) NPs. The resulting solution was filtered through a 0.45 water filter head, followed by dialysis with deionized water for 3 days to remove the organic solvent. Finally, the product was obtained by concentration.

### Synthesis of ^68^Ga-labeled nanoparticles

Elution of ^68^GaCl_3_ from the ^68^Ge/^68^Ga generator with 0.1 M HCl was used for radiolabeling. First, 400 μl ^68^GaCl_3_ (37 MBq), 50 μl PU(L-R-PEG) NPs (1.57 mg) and 100 μl sodium acetate (1.0 M) were mixed, and reacted at different temperatures (30, 60, 90 and 100°C) and different times (5, 15, 30 and 45 min). 0.1 M citric acid was used as solvent to analyze the labeling efficiency by thin layer chromatography (TLC; ScanRAM Radio-TLC Detector LabLogic, UK) [25–27].

The effect of concentration of three kinds of nanoparticles with different rigidity on labeling efficiency was further investigated at the optimum temperature and time. Using 0.1 M citric acid as solvent, the labeling efficiency was analyzed by TLC. All experiments were performed in triplicate.

#### ^68^Ga-labeled small molecule complex

^68^Ga-labeled small molecule complex (^68^GaL) was prepared by heating a mixture of 50 μl L (1.5 mg), 400 μl ^68^GaCl_3_ (37 MBq) and 100 μl sodium acetate (1.0 M) at 100°C for 5 min.

### Stability studies

To explore the labeling stability of ^68^Ga-labeled nanoparticles, PU(^68^Ga-L-R-PEG) NPs (7.4 MBq) were mixed with 0.1 ml of 10% fetal bovine serum or Phosphate Buffer Solution (PBS), respectively. After incubation at 37°C for 30, 60 or 90 min, the labeling efficiency of ^68^Ga was measured by TLC. All experiments were performed in triplicate.

### Establishment of lymph node metastasis model

All the animal experiments were approved by the Experimental Animal Research Committee of Sichuan University. 4T1 cells (5 × 10^4^ cells in 10 μl of 1 × PBS) were injected subcutaneously into the foot pad of the left hind limb of BALB/c mice (Supine position). Hematoxylin–eosin (H&E) staining was used to characterize the successful construction of lymph node metastasis.

### Biodistribution studies

PU(^68^Ga-L-MDI-PEG) NPs (3.7 MBq) were injected into the foot pads of the left and right hind limbs of lymph node metastasis mice (*n* = 4). At 1 and 2 h postinjection, mice were euthanized. Major organs and tissues (blood, heart, liver, spleen, lung, kidney and lymph nodes) of mice were collected. Then, the tissue was weighed and counted on the γ counter (Wizard 2470, Perkinelmer, USA; %ID/g).

### PET/CT imaging

PU(^68^Ga-L-MDI-PEG) NPs (3.7 MBq) were injected into the foot pads of the left and right hind limbs of lymph node metastasis mice (*n* = 4). At 0.5, 1, 1.5 and 2 h postinjection, mice were anesthesized by 2% isoflurane and imaged using a micro-PET/CT scanner (Inviscan, IRIS, Paris, France). The obtained PET images were reconstructed by 3D ordered-subset expectation maximization and then analyzed using Osirix MD software. Regions of interest (ROIs) were manually drawn over the normal lymph nodes (N-LN) and tumor metastatic lymph nodes (T-SLN) and quantitative analyses were calculated. Maximum standardized uptake values (SUV_max_) in ROIs were commonly applied as a quantitative metric.

### Biosafety assessment

*In vitro* cytotoxicity of the PU(L-MDI-PEG) NPs was measured in RAW 264.7 cells by Cell Counting Kit-8 (CCK-8) assay. Around 1 × 10^4^ cells per well were seeded into a 96-well plate overnight and then different concentrations of the PU(L-MDI-PEG) NPs (0, 12.5, 25, 50 and 100 μg/ml) were added for 24 h. Then, the culture medium was discarded. Hundred microliters of fresh medium containing 10% CCK-8 was added to each well and incubated for 2 h. The absorbance of each well was measured at 450 nm on a microplate reader (Thermo Scientific). The cell viability was obtained by the absorbance ratio between the experimental groups and the blank control groups.

Whole blood of BALB/c mice (*n* = 4) was collected by eyeball blood collection in blood collection tubes containing anticoagulants, and then washed with PBS three times to obtain red blood cells (RBCs). Dilute the RBCs (1:10) by adding 100 μl of RBCs to 900 μl of 1 × PBS. PU(L-MDI-PEG) NPs (1.57 mg/ml) were diluted to final concentrations of 12.5, 25, 50 and 100 μg/ml. Diluted RBCs (200 μl) were added to different dilutions of PU(L-MDI-PEG) NPs. The RBCs were incubated at 37°C for 4 h after which were centrifuged at 3000 rpm for 5 min, and then absorbance value was measured at 541 nm using a UV spectrophotometer (Varian Cary 4000).

PU(L-MDI-PEG) NPs (50 μl, 1.57 mg/ml) were injected into normal mice (*n* = 4) through the foot pad. The control group was injected with normal saline. After 24 h of administration, the mice were euthanized, and the whole blood was collected and stood for 4 h. Then, the supernatant was obtained by centrifugation at 3000 rpm for 30 min (4°C), which was used to evaluate the main indexes of liver and kidney function. The mice were then euthanized, and the heart, liver, spleen, lung and kidney of the mice were taken out, washed and fixed with 4% paraformaldehyde, and then the company (Wuhan Saiwei) carried out (H&E) staining.

## Results and discussion

### Preparation and characterization of amphiphilic alternating copolymer ligands

**Figure 1.**
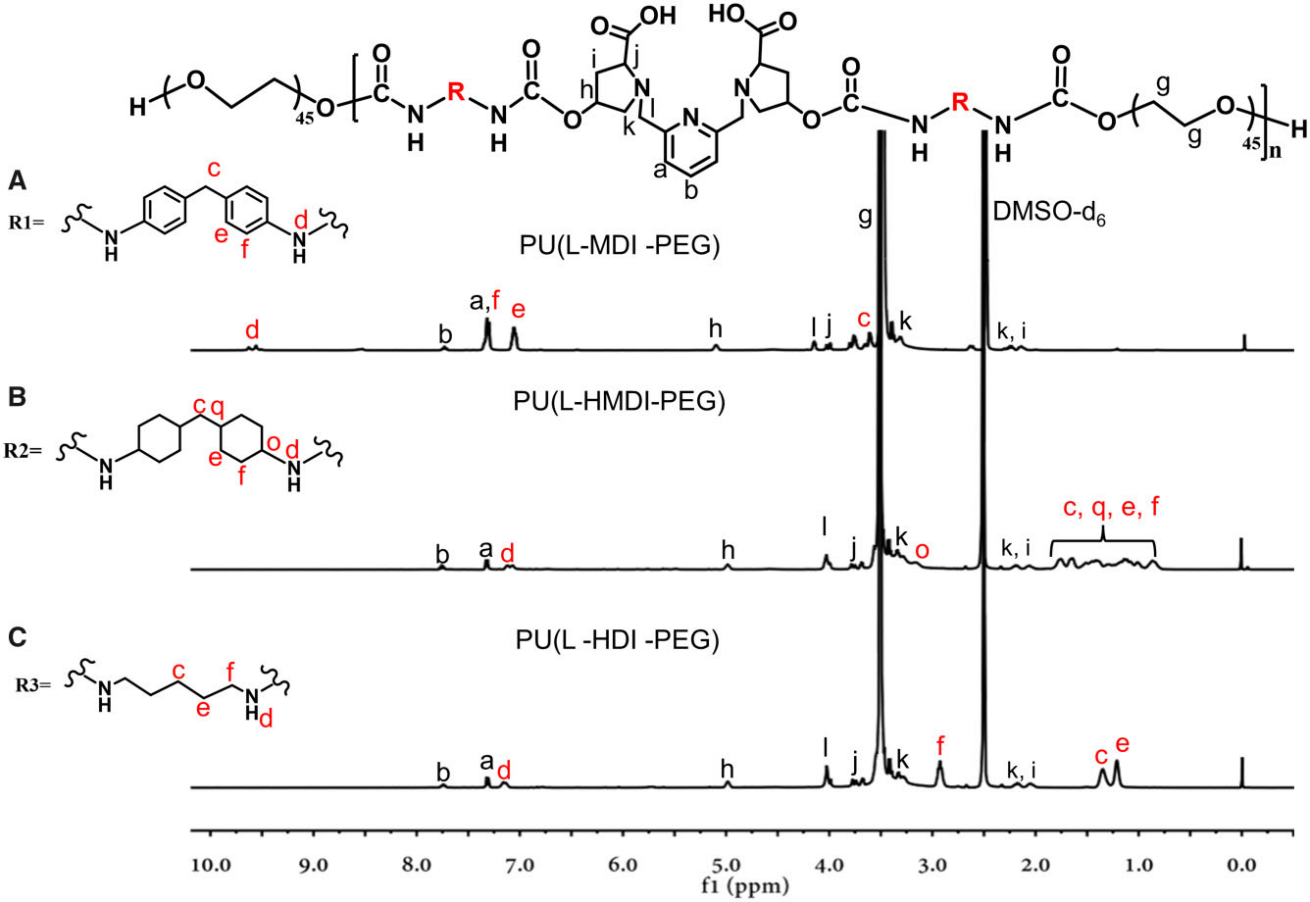
Chemical structure of PU(L-R-PEG) polymer and ^1^H NMR spectra of (**A**) PU(L-MDI-PEG), (**B**) PU(L-HMDI-PEG) and (**C**) PU(L-HDI-PEG) (400 MHz, DMSO-d_6_).

The synthetic route of the amphiphilic alternating copolymer ligands PU(L-MDI-PEG), PU(L-HMDI-PEG) and PU(L-HDI-PEG) was detailed in Supplementary Fig. S1A. The ^1^H NMR results were shown in Fig. 1 and Supplementary Fig. S2. The precursors of PU(L-MDI-PEG), PU(L-HMDI-PEG) and PU(L-HDI-PEG) were synthesized in two steps. Isocyanates of different rigid structures (MDI, HMDI or HDI) were used to form prepolymers with compound 1, followed by polymerization with mPEG_2k_, and finally blocking one end with mPEG_2k_. These precursors were then hydrolyzed in 0.1 M sodium hydroxide to obtain PU(L-MDI-PEG), PU(L-HMDI-PEG) and PU(L-HDI-PEG).

The characteristic peaks of hydrogen protons of mPEG_2k_, three isocyanates (MDI, HMDI and HDI) and compound 1 were visible, indicating that the precursor of PU(L-MDI-PEG), PU(L-HMDI-PEG) and PU(L-HDI-PEG) were successfully synthesized (Supplementary Fig. S1A). Furthermore, in Fig. 1, the methoxy group disappeared (δ 3.59 ppm was attributed to compound 1), demonstrating the successful synthesis of PU(L-MDI-PEG), PU(L-HMDI-PEG) and PU(L-HDI-PEG). The results determined by gel permeation chromatography (GPC; Shimadzu, Japan) demonstrated that the number average molecular mass (Mn) of precursors of PU(L-MDI-PEG), PU(L-HMDI-PEG) and PU(L-HDI-PEG) were 13.3, 10.8 and 10.0 kg/mol with polydispersity indices of 1.98, 1.88 and 1.91, respectively (Supplementary Fig. S3 and Table S1). The molecular weights of PU(L-R-PEG) cannot be acquired by GPC because they were insolvable in most organic solvents such as DMF, CHCl_3_ or THF [28].

**Figure 2.**
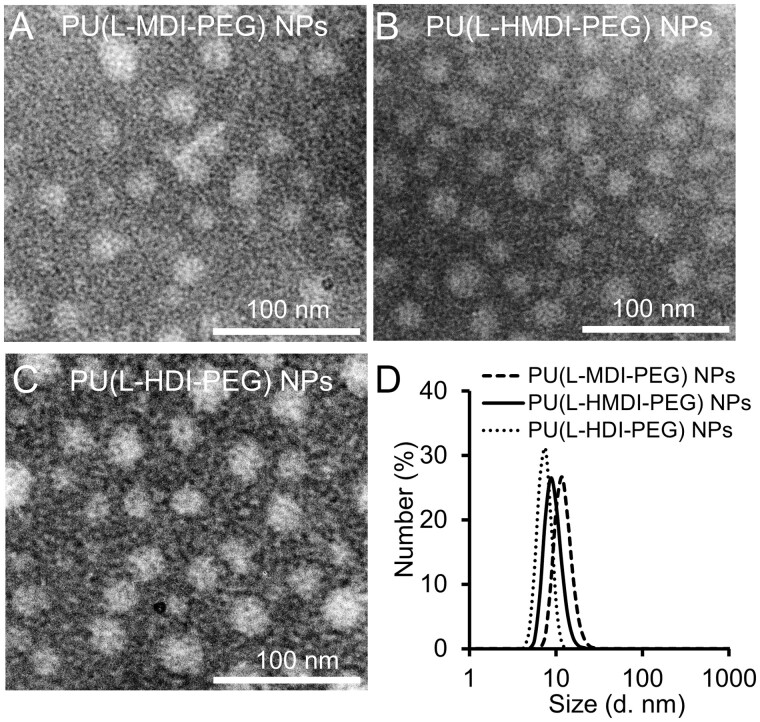
TEM images of (**A**) PU(L-MDI-PEG) NPs, (**B**) PU(L-HMDI-PEG) NPs and (**C**) PU(L-HDI-PEG) NPs. (**D**) Hydrated particle size distribution of PU(L-MDI-PEG) NPs, PU(L-HMDI-PEG) NPs and PU(L-HDI-PEG) NPs.

Compared with water-soluble polymer contrast agents, the stable amphiphilic nanoparticle structure supplies a more rigid platform which can effectively increase stability of chelation [22, 24, 29]. Herein, PU(L-R-PEG) was self-assembled into nanoparticles in water. As measured by the pyrene fluorescent probe method, the critical micelle concentration (CMC) of the PU(L-MDI-PEG), PU(L-HMDI-PEG) and PU(L-HDI-PEG) NPs were 11.2, 20.0 and 18.0 mg/l, respectively (Supplementary Fig. S4) [30–32]. The transmission electron microscopy (TEM; Hitachi H-600, 100 kV) images presented the spherical morphology of PU(L-MDI-PEG), PU(L-HMDI-PEG) and PU(L-HDI-PEG) NPs with an average diameter of 17.7, 17.5 and 17.1 nm, respectively (Fig. 2A–C). The average hydrodynamic diameter of PU(L-MDI-PEG), PU(L-HMDI-PEG) and PU(L-HDI-PEG) NPs were 11.5, 8.7 and 7.5 nm, respectively (Fig. 2D).

### Radiolabeling and stability studies

**Figure 3.**
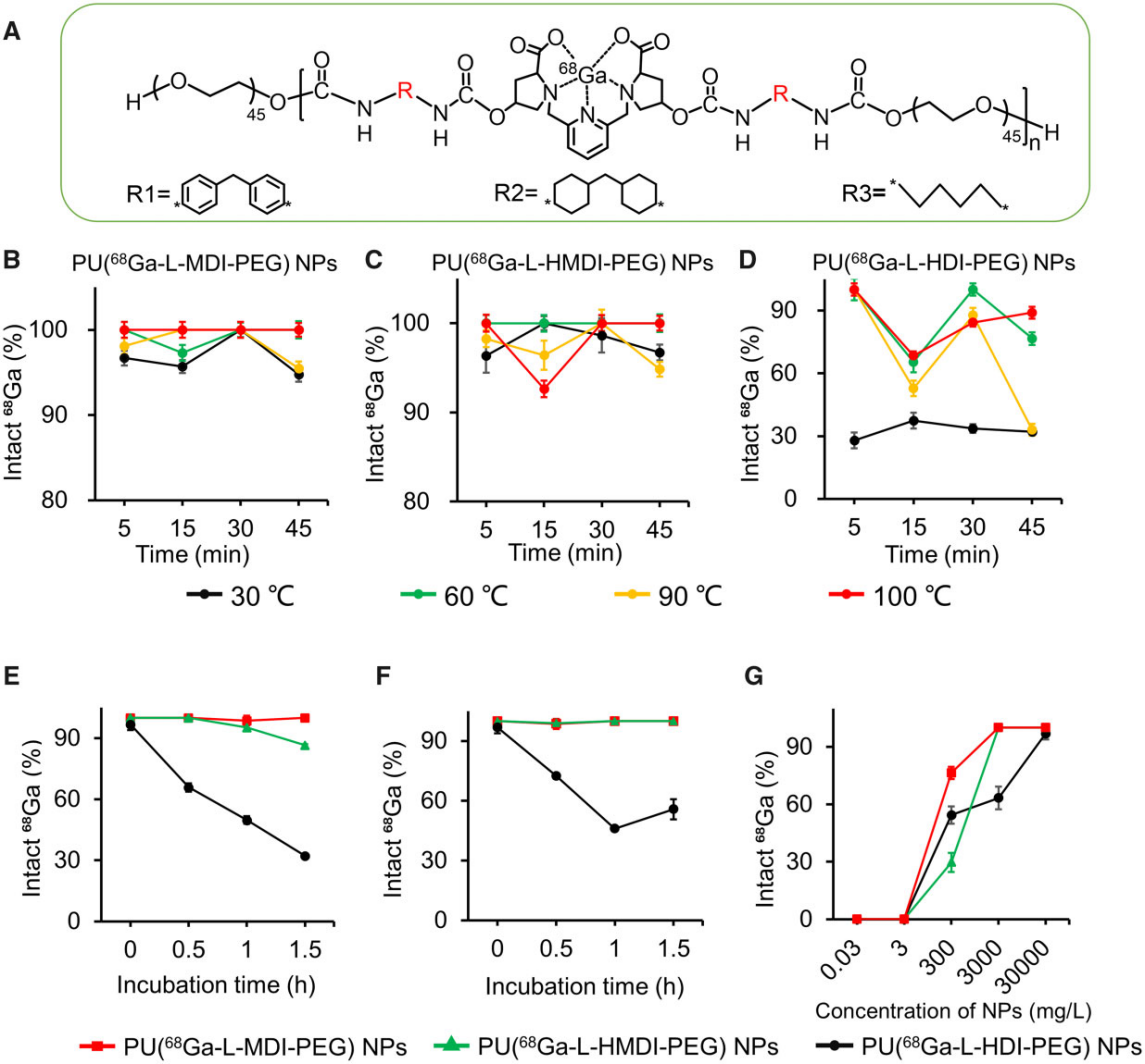
(**A**) Schematic diagram of PU(^68^Ga-L-R-PEG) NPs R = (R1, R2 and R3). optimization of temperature and reaction time of ^68^Ga labeled nanoparticles. Labeling efficiency of (**B**) PU(^68^Ga-L-MDI-PEG) NPs, (**C**) PU(^68^Ga-L-HMDI-PEG) NPs and (**D**) PU(^68^Ga-L-HDI-PEG) NPs at different temperatures and reaction time. The labeling stability of PU(^68^Ga-L-MDI-PEG) NPs, PU(^68^Ga-L-HMDI-PEG) NPs and PU(^68^Ga-L-HDI-PEG) NPs in (**E**) 10% fetal bovine serum and (**F**) PBS, respectively (*n* = 3). (**G**) Effect of concentrations of PU(^68^Ga-L-MDI-PEG) NPs, PU(^68^Ga-L-HMDI-PEG) NPs and PU(^68^Ga-L-HDI-PEG) NPs on ^68^Ga labeling efficiency.

In order to investigate the effect of the rigidity of coordination unit on the ^68^Ga labeling properties of self-assembled nanoparticles, we investigated the effect of the labeling temperature, labeling time and polymer concentration on the labeling stability and the labeling efficiency ^68^Ga-nanoparticles in different media. Figure 3A shows the structural formula of PU(^68^Ga-L-R-PEG) NPs (R = R1, R2 and R3).

First, the effect of temperature (30, 60, 90 and 100°C) on the stability and size of nanoparticles was explored (Supplementary Fig. S5). The experimental results showed that the temperature has little effect on the stability and hydrated particle size of the nanoparticles in the temperature range discussed in this work.

Next, we investigated the labeling temperature and labeling time on labeling efficiency of ^68^Ga-nanoparticles. The results were shown in Fig. 3B and C. First, the labeling efficiency of PU(^68^Ga-L-MDI-PEG) and PU(^68^Ga-L-HMDI-PEG) NPs with strong rigidity coordination units were less affected by temperature and time. These nanoparticles could reach more than 90% labeling efficiency at a wide reaction time and reaction temperature. The optimal radioactive labeling conditions of PU(^68^Ga-L-MDI-PEG) and PU(^68^Ga-L-HMDI-PEG) NPs were 100°C for 5 min and 60°C for 5 min, respectively. The labeling efficiency of PU(^68^Ga-L-MDI-PEG) and PU(^68^Ga-L-HMDI-PEG) NPs was about 98 and 86% after incubation in 10% fetal bovine serum for 1.5 h, and about 100% and 100% in PBS, indicating that they have good labeling stability especially without serum interference (Fig. 3E and F). Second, the labeling efficiency of PU(^68^Ga-L-HDI-PEG) NPs increased significantly with increasing temperature, and its optimal radiolabeling condition was 60°C for 5 min (Fig. 3D). The labeling efficiency of PU(^68^Ga-L-HDI-PEG) NPs was about 32% after incubation in 10% fetal bovine serum for 1.5 h, and about 40% in PBS (Fig. 3E and F). In general, labeling efficiency of PU(^68^Ga-L-HDI-PEG) NPs was greatly affected by temperature and time, and the labeling efficiency was much lower than other two formulations. The above results showed that the increase of rigidity of coordination unit was beneficial to improve the labeling efficiency and stability of nanoparticles, and among them, the highest labeling efficiency and stability of PU(^68^Ga-L-MDI-PEG) NPs may rely on its stronger coordination unit rigidity [33–36].

Finally, the effect of nanoparticle concentration on labeling efficiency was explored. The results showed that with the concentration of polymer nanoparticles increased from 3 × 10^−2^ to 3 × 10^4^ mg/ml, the labeling efficiency increased from 0 to 100% for all three nanoparticles (Fig. 3G). Compared with PU(^68^Ga-L-HMDI-PEG) and PU(^68^Ga-L-HDI-PEG) NPs, PU(^68^Ga-L-MDI-PEG) NPs with stronger rigidity of coordination unit showed higher chelating efficiency at the nanoparticle concentration of 300 mg/ml. It might be that PU(^68^Ga-L-MDI-PEG) NPs had the strongest rigidity and the lowest CMC, which improved the chelation efficiency [33, 37–40]. PU(^68^Ga-L-MDI-PEG) NPs with the best labeling efficiency and stability were used in the following animal experiments, and the optimal radioactive labeling conditions of PU(^68^Ga-L-MDI-PEG) NPs was 100°C for 5 min.

### PET/CT imaging of tumor lymph node metastasis 

Lymph node metastasis has a high incidence in patients with breast cancer, so we constructed an animal model of lymph node metastasis based on murine 4T1 tumor. The H&E staining results of lymph nodes were shown in Supplementary Fig. S6. Compared with the N-LN, T-SLN is significantly enlarged, and there are cells with large nuclei in the indicated sections, suggesting the presence of tumor metastasis [41]. The above results indicated that the 4T1 murine tumor lymph node metastasis model was successfully constructed.

**Figure 4.**
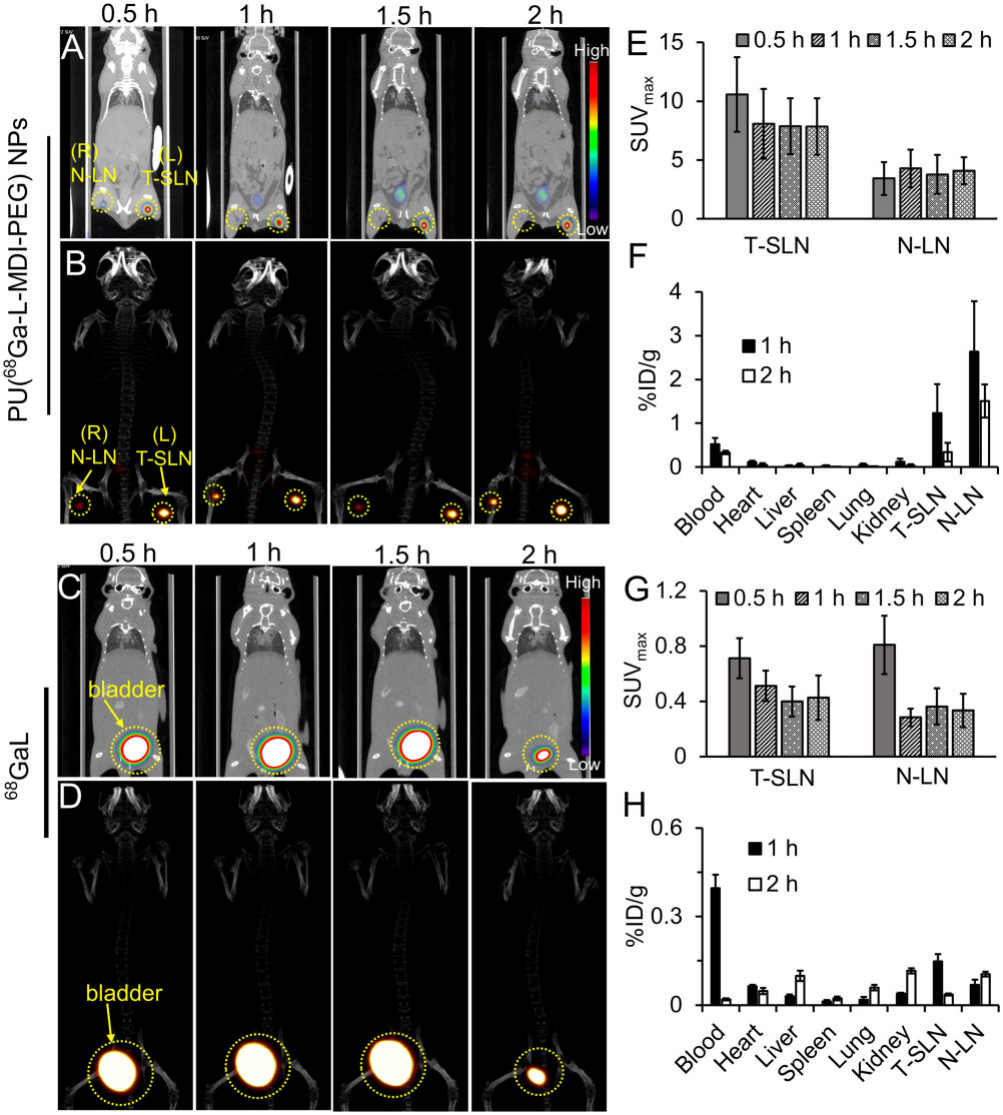
PET imaging of lymph node metastasis. (**A** and **C**) PET/CT fusion images and (**B** and **D**) 3D reconstruction images of lymph node metastasis mice (supine position, *n* = 4) after injection of PU(^68^Ga-L-MDI-PEG) NPs or ^68^GaL (3.7MBq) by footpad injection. SUV_max_ at different time points after injection of (**E**) PU(^68^Ga-L-MDI-PEG) NPs or (**G**) ^68^GaL. Biodistribution of (**F**) PU(^68^Ga-L-MDI-PEG) NPs or (**H**) ^68^GaL in different tissues and organs after 1 and 2 h of injection through footpads (*n* = 4).

The PET imaging results of PU(^68^Ga-L-MDI-PEG) NPs in 4T1 lymph node metastasis mice were shown in Fig. 4A and B. Significant PET signal was observed in left and right lymph nodes through the whole-body scan, indicating that PU(^68^Ga-L-MDI-PEG) NPs successfully accumulated to lymph nodes, which is consistent to previous reports [42–44]. The SUV_max_ of T-SLN was higher than that of N-LN (Fig. 4E). We speculated that the possible reason was that PU(^68^Ga-L-MDI-PEG) NPs were mainly taken up by macrophages, which were abundant in lymph nodes. When the tumor cells invaded the lymph nodes, some macrophages were replaced by tumor cells, resulting in reduced uptake of nanoparticles. However, at the same time, compared with the normal reflux of N-LN, the reflux of T-SLN was blocked, resulting in more PU(^68^Ga-L-MDI-PEG) NPs entrapment in T-SLN and less excretion, which could enhance the PET signal of T-SLN [45, 46]. The biodistribution data for PU(^68^Ga-L-MDI-PEG) NPs was expressed as the percentage of injected dose per gram of tissue in Fig. 4F. Compared with other organs, higher radioactive uptake was found in N-LN and T-SLN at 2 h after injection of PU(^68^Ga-L-MDI-PEG) NPs. Among them, the radioactivity per unit mass of N-LN (2.6% ID/g) was higher than that of T-SLN (1.5% ID/g). The main reason may be explained as the following: although the total radiation dose of T-SLN was higher than that of N-LN, the tumor cells proliferated in T-SLN, making T-SLN 10-15 times larger than N-LN [44, 47]. Therefore, when the radiation amount per unit mass was taken as the measurement standard, the radiation dose of N-LN was higher than that of T-SLN.

**Figure 5.**
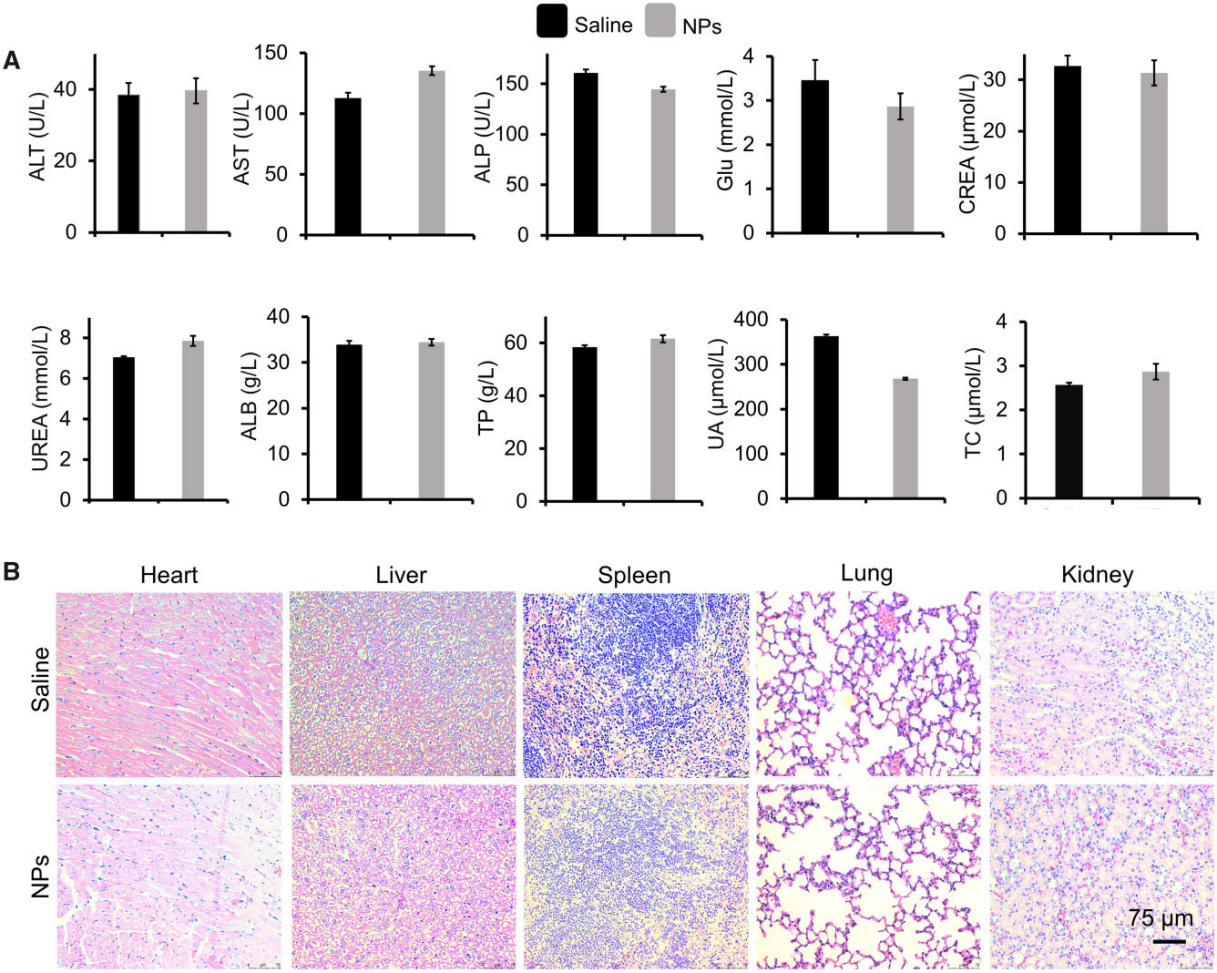
Serum, liver and kidney function indexes and H&E staining pictures. (**A**) The blood biochemical indicators related to liver and kidney function. (**B**) H&E staining pictures of major organs (heart, liver, spleen, lung and kidney). Scale bar: 75 μm.

In comparison, the small molecule ^68^GaL was used as a control group. The synthesis route of ^68^GaL was shown in Supplementary Fig. S1B. The experimental results were shown in Fig. 4C and D. Apparently, the bladder showed much higher PET signal intensity than other organs. But the PET signal intensity of the N-LN and T-SLN was much lower, and no significant difference in SUV_max_ was observed between them (Fig. 5G). This is mainly because contrast agent size is an important factor affecting its absorption and retention in lymph nodes. The small molecule ^68^GaL could easily enter blood vessels and lymphatic capillaries, but the flow rate of blood capillaries was 100–500 times faster than that of lymphatic capillaries [48]. Hence, the small molecule ^68^GaL tended to be cleared from the interstitium through capillaries, circulated through the blood, and was metabolized into urine through the kidney and then excreted from the body [48–50]. The tissue distribution results of ^68^GaL (%ID/g) were shown in Fig. 4H. At 1 h after injection of ^68^GaL, blood radioactivity was significantly higher than that of other organs. Subsequently, ^68^GaL was excreted with the urine, and the radioactivity in the blood decreased significantly. In addition, both N-LN and T-SLN had mild radioactivity enhancement, but there was no significant difference between them (Fig. 4G).

In summary, the small molecule ^68^GaL could not distinguish between N-LN and T-SLN due to the rapid clearance kinetics. PU(^68^Ga-L-MDI-PEG) NPs could successfully distinguish N-LN and T-SLN through PET imaging, and with an extended time window of lymph node imaging.

### Biosafety assessment for PU(L-MDI-PEG) NPs

We performed a preliminary biosafety study of PU(L-MDI-PEG) NPs. The results of cytotoxicity experiments showed that the cell survival rate was higher than 89% after incubation of PU(L-MDI-PEG) NPs with different concentrations (1–100 μg/ml) for 24 h (Supplementary Fig. S7A). Compared with the control group, there was no obvious toxicity in the concentration range of 1–50 μg/ml, while the cell activity was slightly lower than that of the control group at a concentration of 100 μg/ml. It may be that the concentration of polymer nanoparticles is too high, affecting the normal growth of cells. Similar experimental phenomena was also reflected in another report [51]. Then, the hemolysis rate of PU(L-MDI-PEG) NPs with different concentrations was all lower than 0.1%, indicating that PU(L-MDI-PEG) NPs had good blood compatibility (Supplementary Fig. S7B–D). In addition, the systemic toxicity of PU(L-MDI-PEG) NPs (50 μl, 1.57 mg/ml) to healthy mice was investigated by histopathological and biochemical indicators. There was no significant difference in biochemical indexes and pathological sections between PU(L-MDI-PEG) NPs group and the control group (saline), and the indexes were within the normal range, indicating that PU(L-MDI-PEG) NPs group did not cause any hepatorenal toxicity, obvious pathological changes and tissue damage (Fig. 5).

## Conclusions

In this study, the strategies of regulating the rigidity of coordination unit and radiolabeling stability were adopted to prepare the three different ^68^Ga chelating nanoprobes. The results showed that the increasing the rigidity of coordination unit of nanoparticles could reduce their CMC and made them more stable. On the other hand, it was beneficial to improve the labeling efficiency and stability of ^68^Ga labeled nanoparticles. In lymph node metastasis PET imaging, PU(^68^Ga-L-MDI-PEG) NPs could successfully differentiate T-SLN from N-LN. In comparison, small molecule contrast agent ^68^GaL could not differentiate T-SLN from N-LN due to its similar accumulation and rapid metabolism. This work provides a reference for preparation of sensitive PET imaging nanoparticle probes with high labeling efficiency and stability, and potential application in lymph node metastasis imaging.

## Supplementary Material

rbad029_Supplementary_Data
