# Supplementary material for: 68Ga-labeled amphiphilic polymer nanoparticles for PET imaging of sentinel lymph node metastasis
Source: Regen Biomater. 2023 Mar 27;10:rbad029. doi: 10.1093/rb/rbad029 (PMC10112949; doi:10.1093/rb/rbad029)
Supplement: rbad029_Supplementary_Data [file rbad029_supplementary_data.zip › 20230315-supporting.docx]

**Supporting information**

**^68^Ga-labeled amphiphilic polymer nanoparticles for PET imaging of sentinel lymph node metastasis**

Qin Chen^1#^, Xiaomin Fu^1#^, Huawei Cai^3#^, Shengxiang Fu^1^, Zhongyuan Cai^1^, Mufeng Li^3^, Xiaoai Wu^3^, Rong Tian^3^, Hua Ai^1, 2*^

1. National Engineering Research Center for Biomaterials, Sichuan University, Chengdu 610064, China
2. Department of Radiology, West China Hospital, Sichuan University, Chengdu 610041, China
3. Department of Nuclear Medicine, West China Hospital, Sichuan University, Chengdu, 610041, China

# The authors contribute equally to the work.

* Corresponding author:

Hua Ai, National Engineering Research Center for Biomaterials, Sichuan University, Chengdu 610064, P. R. China Phone: 86-28-85413991, Email: huaai@scu.edu.cn


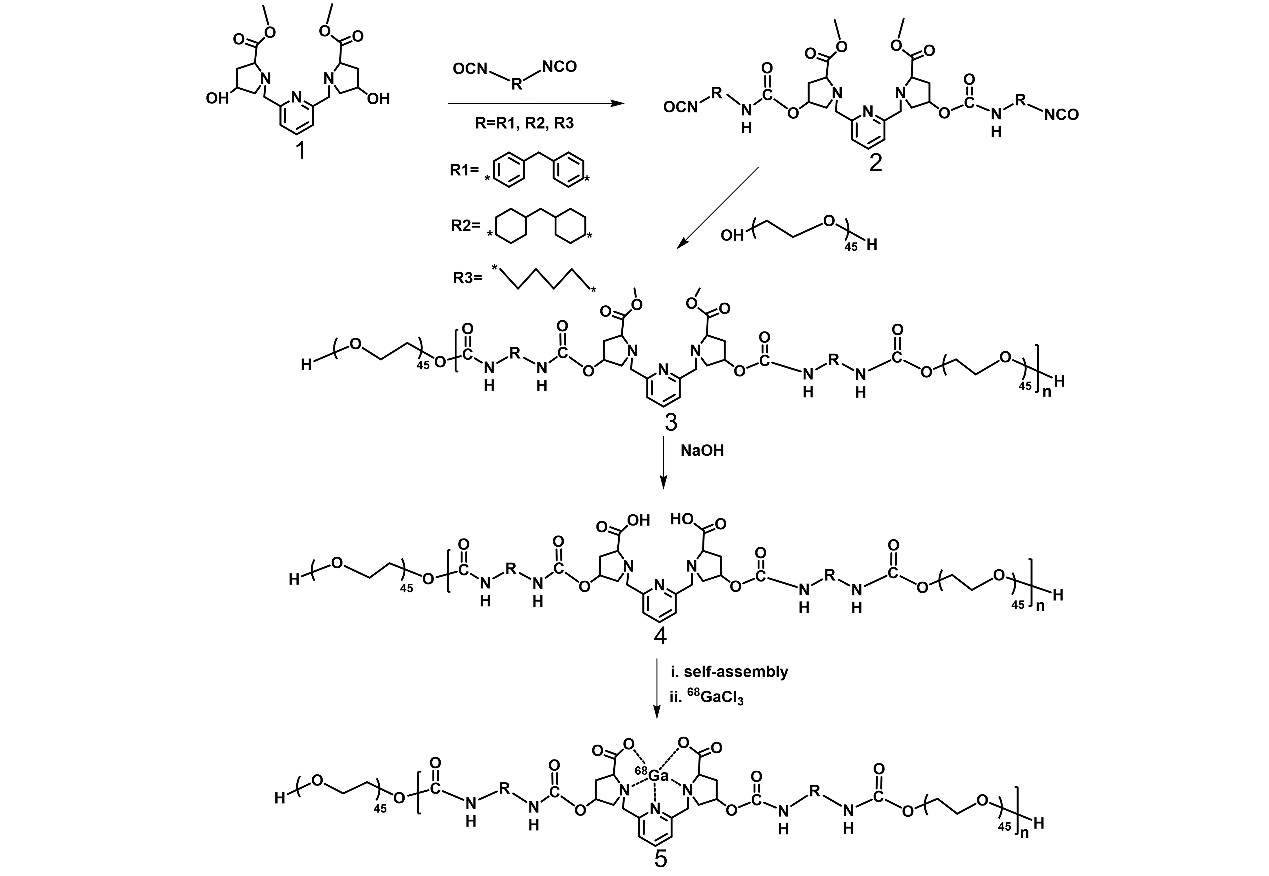


**Figure S1.** Synthetic route of PU(^68^Ga-L-R-PEG) NPs. R = (R1, R2 and R3)


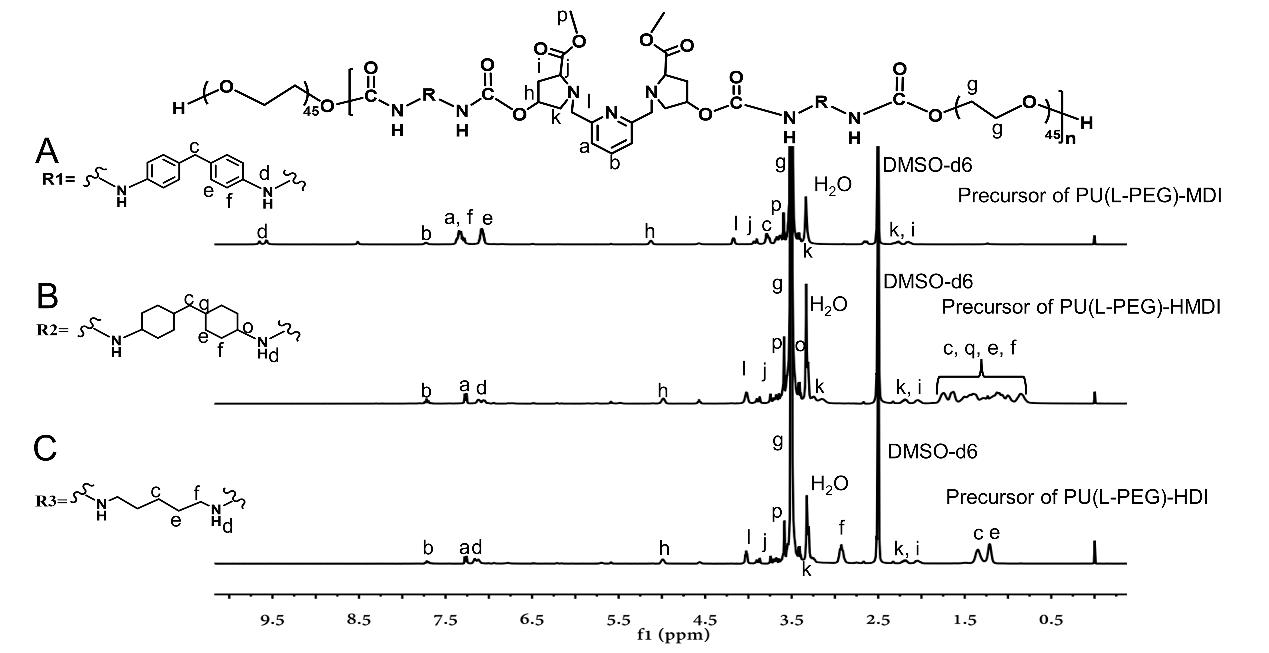


**Figure S2.** ^1^H NMR spectra of (A) precursor of PU(L-MDI-PEG), (B) precursor of PU(L-HMDI-PEG) and (C) precursor of PU(L-HDI-PEG).


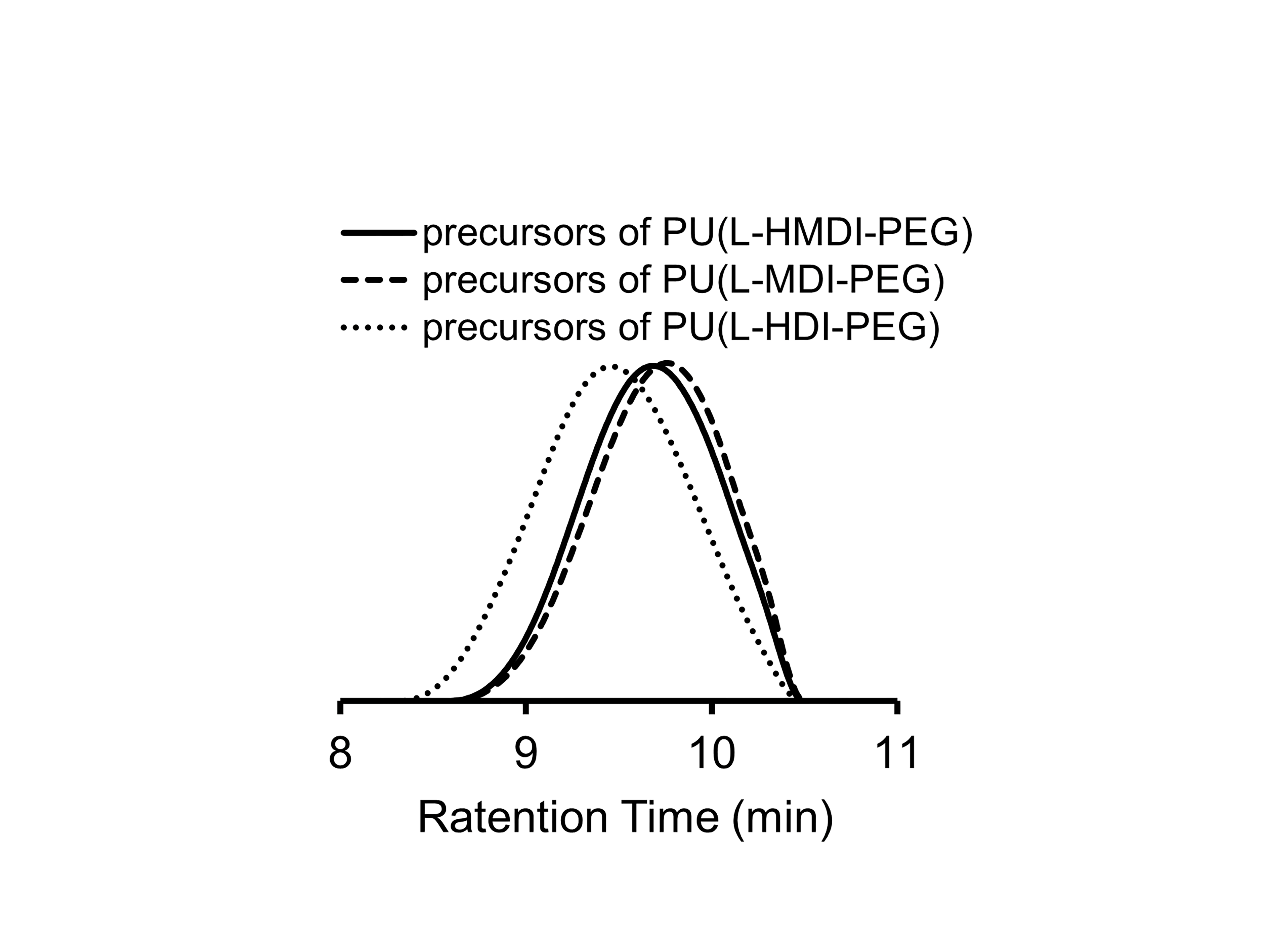


**Figure S3.** GPC of the precursors of PU(L-MDI-PEG), PU(L-HMDI-PEG) and PU(L-HDI-PEG). The mobility solvent was DMF.

**Table S1.** The molecular weight of the precursors of PU(L-MDI-PEG), PU(L-HMDI-PEG) and PU(L-HDI-PEG).


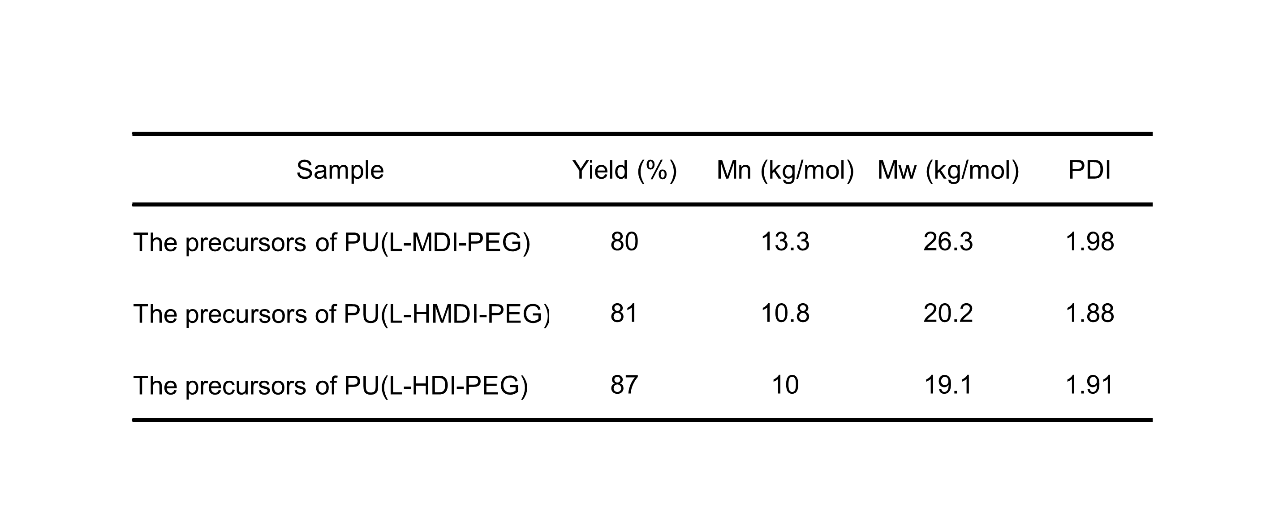


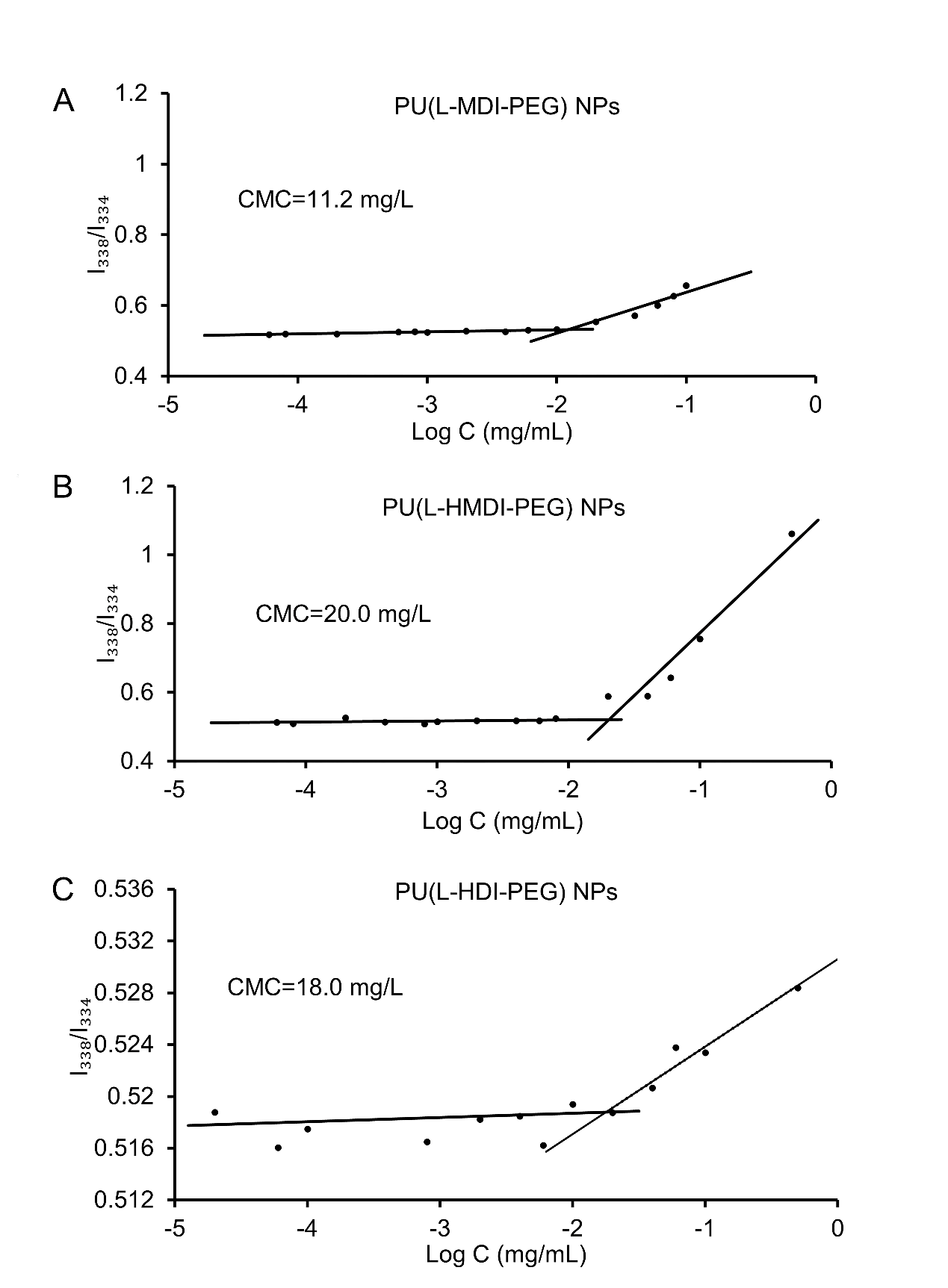


**Figure S4.** CMC of PU(L-MDI-PEG) NPs, PU(L-HMDI-PEG) NPs and PU(L-HDI-PEG) NPs


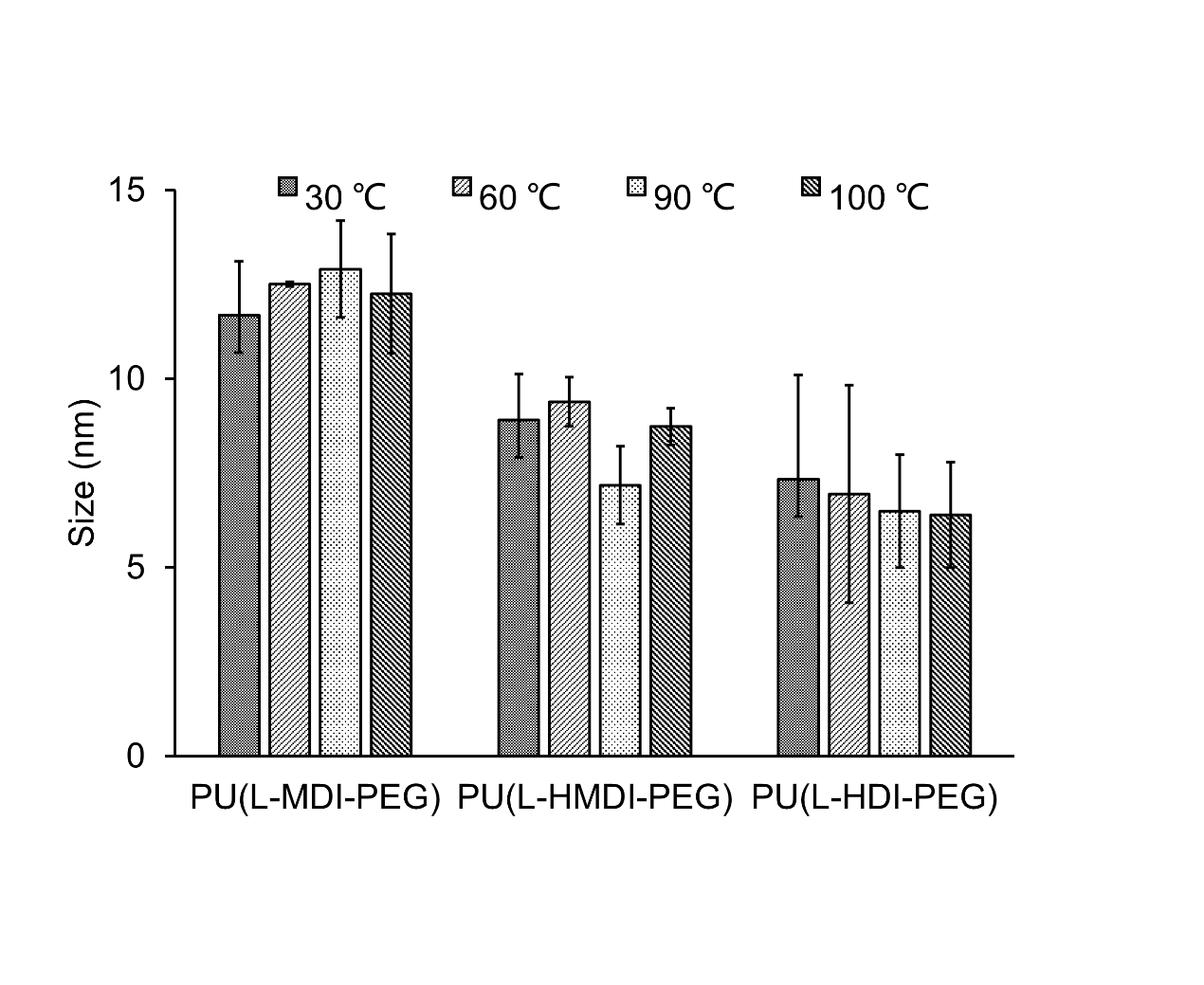


**Figure S5.** At different temperatures hydrated particle size distribution of PU(L-MDI-PEG) NPs, PU(L-HMDI-PEG) NPs and PU(L-HDI-PEG) NPs.


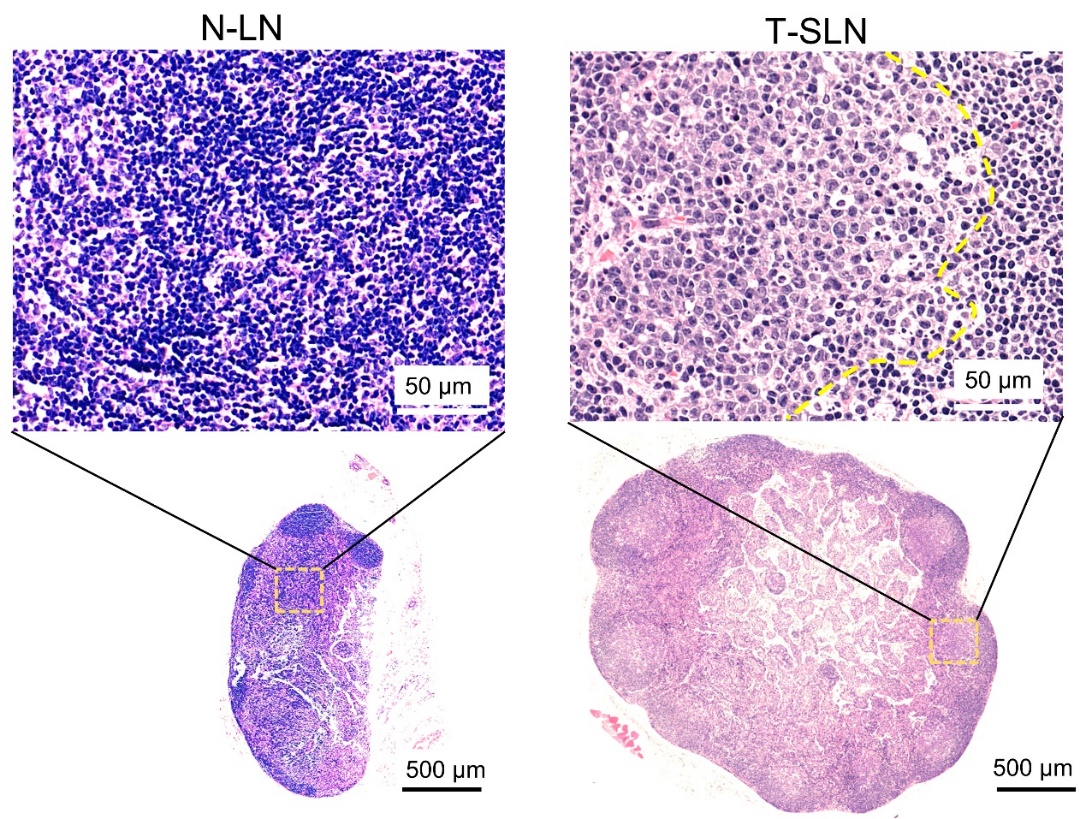


**Figure S6.** H&E staining for the N-LN and T-SLN.


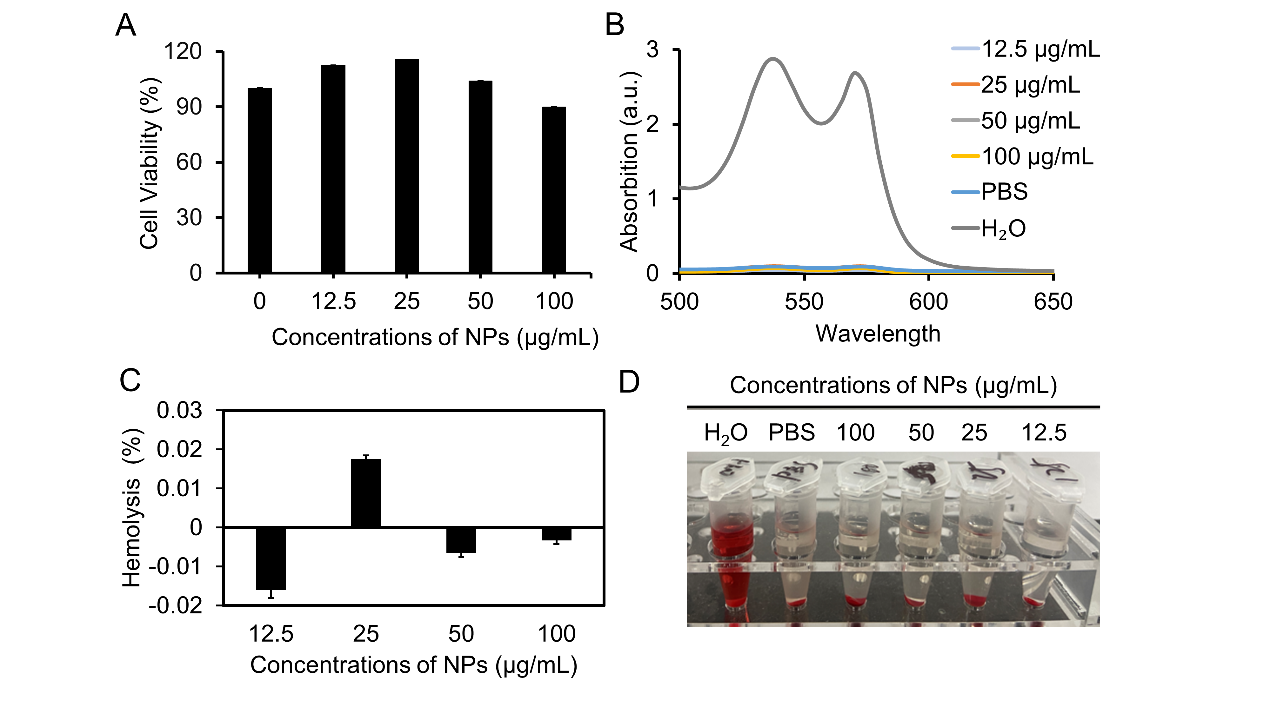


**Figure S7.** (A) Relative cell viability (%) of Raw264.7 cells incubated with PU(L-MDI-PEG) NPs for 24 h. (B) The visible absorption of blood treated with water, PBS, and PU(L-MDI-PEG) NPs. (C) The hemolysis (%) of PU(L-MDI-PEG) NPs. (D) Photographs of the hemolysis of RBCs after being incubated with water, PBS and PU(L-MDI-PEG) NPs, respectively.
